# A Shift in Central Metabolism Accompanies Virulence Activation in Pseudomonas aeruginosa

**DOI:** 10.1128/mBio.02730-18

**Published:** 2020-03-10

**Authors:** Kumar Perinbam, Jenu V. Chacko, Anerudh Kannan, Michelle A. Digman, Albert Siryaporn

**Affiliations:** a Department of Physics and Astronomy, University of California, Irvine, Irvine, California, USA; b Department of Biomedical Engineering, University of California, Irvine, Irvine, California, USA; c Department of Molecular Biology and Biochemistry, University of California, Irvine, Irvine, California, USA; University of California, San Francisco

**Keywords:** FLIM, PilY1, antivirulence, central metabolism, fluorescence lifetime imaging microscopy, quorum sensing, surface sensing, virulence regulation

## Abstract

The availability of energy has significant impact on cell physiology. However, the role of cellular metabolism in bacterial pathogenesis is not understood. We investigated the dynamics of central metabolism during virulence induction by surface sensing and quorum sensing in early-stage biofilms of the multidrug-resistant bacterium Pseudomonas aeruginosa. We established a metabolic profile for P. aeruginosa using fluorescence lifetime imaging microscopy (FLIM), which reports the activity of NADH in live cells. We identified a critical growth transition period during which virulence is activated. We performed FLIM measurements and direct measurements of NADH and NAD^+^ concentrations during this period. Here, planktonic (low-virulence) and surface-attached (virulence-activated) populations diverged into distinct metabolic states, with the surface-attached population exhibiting FLIM lifetimes that were associated with lower levels of enzyme-bound NADH and decreasing total NAD(H) production. We inhibited virulence by perturbing central metabolism using citrate and pyruvate, which further decreased the enzyme-bound NADH fraction and total NAD(H) production and suggested the involvement of the glyoxylate pathway in virulence activation in surface-attached populations. In addition, we induced virulence at an earlier time using the electron transport chain oxidase inhibitor antimycin A. Our results demonstrate the use of FLIM to noninvasively measure NADH dynamics in biofilms and suggest a model in which a metabolic rearrangement accompanies the virulence activation period.

## Abstract (precis)

The rise of antibiotic resistance requires the development of new strategies to combat bacterial infection and pathogenesis. A major direction has been the development of drugs that broadly target virulence. However, few targets have been identified due to the species-specific nature of many virulence regulators. The lack of a virulence regulator that is conserved across species has presented a further challenge to the development of therapeutics. Here, we identify that NADH activity has an important role in the induction of virulence in the pathogen P. aeruginosa. This finding, coupled with the ubiquity of NADH in bacterial pathogens, opens up the possibility of targeting enzymes that process NADH as a potential broad antivirulence approach.

## INTRODUCTION

Pseudomonas aeruginosa is an opportunistic pathogen that is responsible for a range of illnesses, including lung infections in cystic fibrosis patients and hospital-acquired infections, sepsis, and disease in immunocompromised patients (1). The bacterium infects a broad range of hosts, including humans, animals, plants, insects, amoebae, and other bacteria using a multitude of virulence factors, including the type III secretion system, cyanide, pyocyanin, and proteases (2–5). Recent work has reported that the expression of virulence factors in P. aeruginosa is regulated by nutrient availability and central metabolic networks (6–8). In addition, tricarboxylic acid cycle (TCA) intermediates alter the activity of some virulence factor regulators in Gram-positive and intracellular pathogens (9, 10). These studies provide a static snapshot of the involvement of metabolism in virulence regulation. The dynamics of central metabolic activity during the activation of virulence in P. aeruginosa are not known, and many questions remain about the regulatory link between central metabolism and virulence activation. What are the energetic requirements for the expression of virulence factors? Can central metabolism be tuned to inhibit virulence? We investigated these questions by measuring central metabolic activity during the transition from a low-virulence state to an activated virulence state in P. aeruginosa.

Virulence factor production is induced in P. aeruginosa and other bacteria through the activation of surface sensing (11–15). The host-killing mechanism of surface-activated virulence in P. aeruginosa has not been attributed to a single virulence factor, including a type III secretion product, pyocyanin, or elastase, but has been attributed to the combinatorial nature of virulence factor production (2, 12). A recent preprint reports that alkyl quinolones are a critical cytotoxic factor (16). Virulence induction by surface attachment is dependent on the protein PilY1, which is found on the outer surface of the cell membrane, contains homology to a mechanically active von Willebrand factor domain, mediates a c-di-GMP response to shear stress, and is required for the initiation of biofilm formation (17–21). Surface-induced virulence also requires coactivation of quorum sensing, which is triggered when cells reach a threshold density (22, 23). Surface sensing and quorum sensing form a coincidence gate in which the activation of both pathways is required to induce virulence (12).

Surface attachment regulates the levels of the metabolites cyclic AMP and cyclic di-GMP (13, 20, 21, 24) and upregulates transcription of NADH-associated enzymes (12). Quorum sensing, which controls the expression of many virulence factors, produces a major shift in the production of a large fraction of metabolites (25). It is possible that surface sensing and quorum sensing induce virulence through changes in central metabolism. However, addressing this hypothesis has been challenging because surface sensing and quorum sensing are dynamic processes and monitoring their effects requires simultaneous measurements of both virulence and central metabolism.

The phasor approach to fluorescence lifetime imaging microscopy (FLIM) measures the dynamics of central metabolism in live cells. This method reports the relative abundance of the free and bound forms of NADH by exploiting its autofluorescent properties (26, 27). This method has been used extensively to track changes in NADH forms in live eukaryotic cells during critical cell processes, including duplication, proliferation, and differentiation (28–32). NADH is excited using two-photon excitation and decays to the ground state with distinct decay rates, or lifetimes, in the visible spectrum. The major advantage of this approach is the ability to track spatial and temporal changes in metabolic activity at subcellular resolution without the need to label molecules, introduce fluorescent reporters, or to stain, perturb, or harvest cells. Advances in optics, imaging, and analysis have enabled fluorescence lifetime measurements in a number of bacteria, including Lactobacillus acidophilus, Escherichia coli, and P. aeruginosa (33–37). However, independent measurements of NADH were not performed in these studies, which limited the interpretation of the FLIM measurements. In addition, the FLIM measurements were not performed during virulence activation.

Here, we establish a metabolic trajectory in P. aeruginosa using FLIM and through independent *in vitro* measurements of NADH and NAD^+^ concentrations. We measure metabolic states in P. aeruginosa during a critical growth transition in which virulence is activated in surface-attached cells. We show that compared to low-virulence (planktonic) cells, virulence-activated (surface-attached) cells exhibit FLIM lifetimes that are associated with decreased levels of enzyme-bound NADH and decreased NAD(H) production. Perturbation of central metabolism using citrate or pyruvate, which further decreases enzyme-bound NADH and total NAD(H) production, inhibits virulence, while treatment using an electron transport chain oxidase inhibitor induces virulence at an earlier time.

## RESULTS

### Fluorescence lifetimes shift with changes in NAD(H) production in P. aeruginosa.

**FIG 1:**
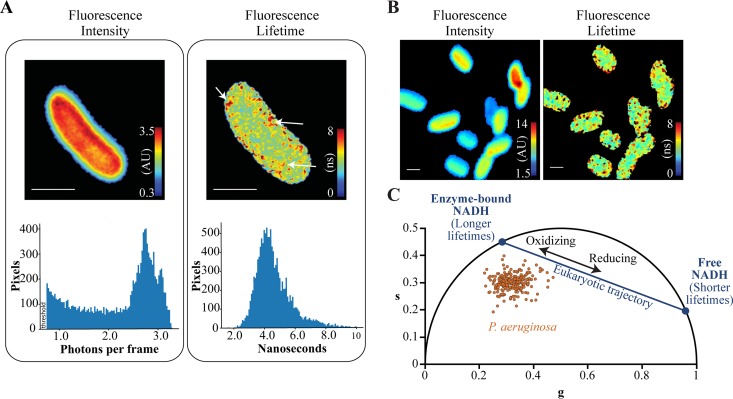
Metabolic profiling of live planktonic P. aeruginosa cells using fluorescence lifetime imaging microscopy. (A) Fluorescence intensities (in arbitrary units [AU]) and fluorescence lifetimes (in nanoseconds) of an unlabeled P. aeruginosa cell imaged using the NADH emission spectrum. The corresponding fluorescence intensity and lifetime histograms are displayed below the images. Arrows indicate clusters with relatively long fluorescence lifetimes. Bars, 1 μm. (B) Fluorescence intensities and lifetimes of multiple P. aeruginosa cells. Bars, 1 μm. (C) Phasor plot in which the cosine and sine components of the fluorescence lifetime are transformed into *g* and *s* coordinates, respectively. Each dot represents the fluorescence lifetime averaged over an individual P. aeruginosa cell. The metabolic trajectory of eukaryotic cells is plotted for reference using lifetime values of 0.4 ns and 3.4 ns for free and protein-bound NADH, respectively. Planktonic P. aeruginosa from three independent experiments were cultured to early exponential phase in modified minimal medium containing 0.2% citrate as the carbon source.

We measured the fluorescence intensities and lifetimes of unlabeled wild-type P. aeruginosa using two-photon microscopy with excitation at 740 nm and emission centered at 460 nm with an 80-nm bandwidth (NADH emission spectrum). Studies of eukaryotic cells have established that fluorescence intensity is proportional to the concentration of intracellular NADH or NADPH and that fluorescence lifetime is determined by the relative fraction of NADH or NADPH that is free or bound to proteins (28–31, 38–41). The fluorescence lifetimes of NADH and NADPH are indistinguishable using the techniques in this study, and thus, we do not differentiate between these species and refer to NADH only. We cultured P. aeruginosa in minimal medium and measured fluorescence at mid-exponential phase. Fluorescence intensities were uniformly distributed in the cytoplasmic region of the cell and dropped off sharply at the periphery (Fig. 1A and B). In contrast, fluorescence lifetimes were relatively heterogeneous within the cytoplasm, and longer fluorescence lifetimes were localized to multiple clusters within the cell (Fig. 1A and B).

We analyzed fluorescence lifetimes using the phasor approach, which determines the relative fraction of free and enzyme-bound forms of NADH and is insensitive to the total intracellular concentration of NADH (26, 27). In this approach, fluorescence decay curves are transformed by cosine and sine functions and plotted along *g* and *s* axes, respectively. Fluorescence lifetimes that arise from a single species map to the “universal circle” of the phasor (26, 27) (black semicircle in Fig. 1C). Free (unbound) NADH has a relatively short lifetime (0.4 ns) and maps to a coordinate on the lower right region of the universal circle (Fig. 1C). NADH molecules that are bound to eukaryotic enzymes have longer lifetimes (3.2 to 9 ns) and map to coordinates on the upper left region of the universal circle (28, 29, 39, 40) (Fig. 1C). The positions of free and enzyme-bound NADH on the universal circle serve as reference points. Lifetime decay curves that are generated by a mixture of free and enzyme-bound NADH species, which is typically observed in fluorescence lifetime measurements that are averaged over entire eukaryotic cells, map along a linear trajectory that connects the two reference points on the universal circle (28, 29, 40) (Fig. 1C).

Previous studies of eukaryotic cells demonstrate that growth under oxidizing conditions, which decreases the relative free NADH pool and the ratio of free to enzyme-bound NADH, results in increased fluorescence lifetimes and phasor positions that are further away from the free NADH reference point (28–30) (Fig. 1C). Conversely, growth in a reducing environment or blocking oxidative phosphorylation through metabolic inhibitors, both of which increase the relative free NADH pool, results in decreased lifetimes and phasor positions that are closer to the free NADH reference (28–30) (Fig. 1C). These studies establish that the position of lifetimes along the linear trajectory is an indicator of the relative abundance of free and enzyme-bound NADH within the cell.

**FIG 2:**
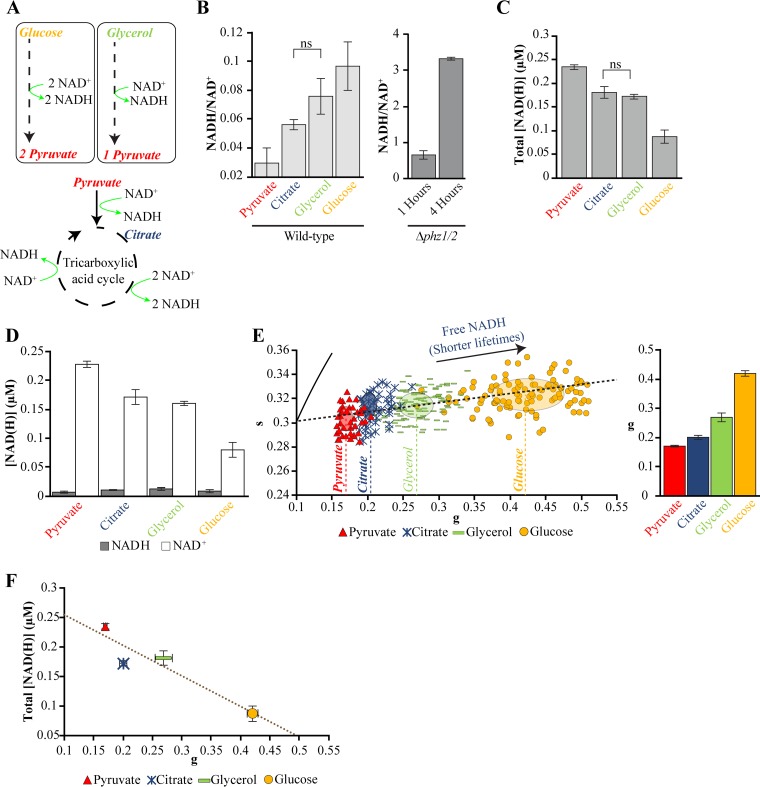
Carbon sources alter NAD(H) concentrations and fluorescence lifetimes in surface-attached P. aeruginosa. (A) Schematic indicating the production of NADH by central metabolism pathways in P. aeruginosa. (B to D) Measurements of NADH/NAD^+^ ratios (B), total NADH and NAD^+^ production (C), and the concentration of NADH or NAD^+^ measured using an enzyme-cycling assay using surface-attached P. aeruginosa cultured using single carbon sources at 0.2% (D). ns, not significant. Bars indicate the average values and error bars indicate the standard deviations from three independent experiments. (E) Fluorescence lifetime phasor plot (left) and corresponding *g* values (right) of P. aeruginosa cultured using identical conditions as the enzyme-cycling assay. Points on the map represent the average fluorescence lifetime of approximately five cells. The centers and axis lengths of the ellipses correspond to the mean and standard deviation, respectively, of all cells pooled from three independent experiments. The dashed line indicates a metabolic trajectory that connects the average phasor positions for different carbon sources. The arrow indicates the direction toward the position of the free NADH reference, which does not appear on this plot. The bars and error bars in the bar graph indicate the means and standard deviations of the means from three independent experiments, respectively. (F) Total NAD(H) concentrations and fluorescence lifetime *g* values for growth on single carbon sources. The points and error bars indicate the means and standard deviations of the means from three independent experiments, respectively. The best-fit line is indicated with *R* = −0.94. All measurements were made on surface-attached P. aeruginosa cells that were cultured in modified minimal medium and harvested at mid-exponential phase, with the exception of the Δ*phz1/2* strain, which was cultured in modified MOPS medium and harvested at different phases. Full statistical details are given in Table S1 in the supplemental material.

In our experiments, P. aeruginosa cells mapped outside of the eukaryotic NADH trajectory (Fig. 1C). The central metabolic enzymes that bind NADH in P. aeruginosa and eukaryotes are distinct (42, 43), and thus, there is no expectation that fluorescence lifetimes for eukaryotes and bacteria should appear along the same trajectory. To establish a metabolic trajectory for P. aeruginosa, we cultured cells in minimal media supplemented with single carbon sources that are associated with different levels of NADH production. Citrate is metabolized through the tricarboxylic acid cycle (TCA) cycle, which reduces NAD^+^ to NADH (Fig. 2A). Glycerol and glucose are metabolized through the Entner-Doudoroff pathway, which produces NADH molecules and pyruvate, the latter of which can enter the TCA cycle (42–44). We expected that the metabolism of citrate, glycerol, glucose, or pyruvate would produce different levels of NADH (Fig. 2A).

10.1128/mBio.02730-18.7 TABLE S1 Supporting statistical data. *t* values, degrees of freedom, and *P* values for unpaired two-tailed heteroscedastic *t* tests that appear in the main figures and supplemental figures. Download Table S1, PDF file, 0.06 MB. Copyright © 2020 Perinbam et al. 2020 Perinbam et al. This content is distributed under the terms of the Creative Commons Attribution 4.0 International license.

We quantified the concentrations of intracellular NADH and NAD^+^ in surface-attached cells that were cultured in each of the carbon sources (Fig. 2B to D) using an enzyme-cycling-based colorimetric assay (45, 46). This assay serves as an independent approach to measuring changes in NADH in addition to the FLIM technique. We observed that the assay was sensitive to the concentration of P. aeruginosa and therefore normalized samples by total protein concentration. We included measurements of a Δ*phz1/2* strain, which does not produce pyocyanin, to identify the protein concentration required to measure changes in NADH and NAD^+^. The NADH/NAD^+^ ratio of this strain increases significantly during the transition from early exponential to late exponential phase (46). The observed increase in ratio between these growth phases (Fig. 2B) established that the P. aeruginosa concentrations in our samples were within the dynamic range of the assay.

Consistent with expectations, we observed that each carbon source yielded a different NADH/NAD^+^ ratio (Fig. 2B). In particular, pyruvate produced the smallest ratio, whereas citrate, glycerol, and glucose yielded increasing ratios. The total concentrations of NADH and NAD^+^ were highest for pyruvate, intermediate for citrate and glycerol, and smallest for glucose (Fig. 2C). The changes in the NADH/NAD^+^ ratios and total concentrations of NAD(H) were primarily due to changes in NAD^+^ production, as the NADH concentrations were relatively constant (Fig. 2D).

We hypothesized that the changes in NADH/NAD^+^ ratios and NAD(H) concentrations are accompanied by changes in the relative fractions of enzyme-bound and free NADH, which would be observed as changes in fluorescence lifetime. We measured fluorescence lifetimes (at single-cell resolution) of surface-attached P. aeruginosa that were cultured under the same conditions as the NAD(H) measurements. We observed significant changes in fluorescence lifetimes primarily along the *g* axis (Fig. 2E). Growth using pyruvate yielded the smallest *g* value, whereas growth using citrate, glycerol, and glucose yielded increasing *g* values (Fig. 2E). Interestingly, *g* values anticorrelated (negatively correlated) with total NAD(H) concentrations (Fig. 2F) (*R* = −0.94). To our knowledge, this relationship has not been identified previously for P. aeruginosa.

We repeated NAD(H) and fluorescence lifetime measurements using planktonic cells that were isolated from the same cultures as the surface-attached cells. The trends in NADH/NAD^+^ ratios and total NAD(H) concentrations for these cells (see Fig. S1A to C in the supplemental material) were identical to those of surface-attached cells (Fig. 2B to D) with the exception that in planktonic cells, citrate produced a slightly greater NADH/NAD^+^ ratio and slightly lower NAD(H) concentration than glycerol (Fig. S1A and B). However, the differences in NADH/NAD^+^ ratios and total NAD(H) concentrations using citrate or glycerol as a carbon source in surface-attached cells were not significant (Fig. 2B and C). The trend in fluorescence lifetime *g* values in planktonic cells (Fig. S1D) was identical to that of surface-attached cells (Fig. 2E). Fluorescence lifetime *g* values and total NAD(H) concentrations in planktonic cells were also anticorrelated (Fig. S1E, *R* = −0.86). These results indicate that the impacts of carbon sources on FLIM and NAD(H) production were qualitatively comparable for both planktonic and surface-attached P. aeruginosa. We note that the changes in NAD(H) concentrations in planktonic cells arose from changes in both NADH and NAD^+^ (Fig. S1C), in contrast to surface-attached cells, where changes in only NAD^+^ were observed (Fig. 2D).

10.1128/mBio.02730-18.1 FIG S1 NAD(H) ratios and concentrations and fluorescence lifetime *g* values of planktonic P. aeruginosa cultured using single carbon sources. (A) NADH/NAD^+^ ratios, (B) total NADH and NAD^+^ concentrations, (C) individual NADH and NAD^+^ concentrations, and (D) fluorescence lifetime phasor values of planktonic wild-type P. aeruginosa that were cultured using single carbon sources. (D) Fluorescence lifetime phasor plot (left) and corresponding *g* values (right). Points on the map represent the average fluorescence lifetime of approximately five cells. The center and axis length of each ellipse corresponds to the mean and standard deviation, respectively, of all cells pooled from three independent experiments. The arrow indicates the direction toward the position of the free NADH reference, which does not appear on this plot. The bars and error bars in the bar graph indicate the means and standard deviations of the means from three independent experiments, respectively. The dashed line indicates a metabolic trajectory that connects the average phasor positions for different carbon sources. (E) Total NAD(H) concentrations and fluorescence lifetime *g* values plotted together. The best-fit line is indicated with *R* = −0.86. The points and error bars indicate the means and standard deviations of the means from three independent experiments, respectively. Planktonic P. aeruginosa cells were cultured in modified minimal medium and harvested at mid-exponential phase at an OD_600_ of 0.2 with the exception of the Δ*phz1/2* strain, which was cultured in modified MOPS medium and harvested at 1 and 4 h following dilution. *, *P* < 0.05. Full statistical details are given in Table S1 in the supplemental material. Download FIG S1, PDF file, 0.1 MB. Copyright © 2020 Perinbam et al. 2020 Perinbam et al. This content is distributed under the terms of the Creative Commons Attribution 4.0 International license.

The relative shifts in fluorescence lifetime are not due to pyoverdine or pyocyanin, which are fluorescent molecules produced in high abundance by P. aeruginosa (46, 47), because the lifetime shifts were observed in strains that are defective in the production of pyoverdine or pyocyanin (see Fig. S2A and B in the supplemental material). Purified forms of pyoverdine and pyocyanin also mapped to positions outside the P. aeruginosa lifetime range (Fig. S2C). Furthermore, a shift toward higher *g* value (greater fraction of free NADH) was observed when P. aeruginosa was treated with the oxidase inhibitor antimycin A, which inhibits electron transport chain activity (48, 49) (Fig. S2D). Together, these results establish a fluorescence lifetime metabolic activity trajectory for P. aeruginosa (Fig. 2E and Fig. S1D). In particular, the P. aeruginosa trajectory is positioned below the eukaryotic metabolic trajectory (Fig. S2C) on the phasor diagram, and the shifts are mostly along the *g* axis. These data indicate that the selection of carbon source affects total NAD(H) production and the relative fractions of free and enzyme-bound NADH. The observation that changes in NAD(H) production are anticorrelated with changes in fluorescence lifetime (Fig. 2F and Fig. S1E) suggest that NAD(H) production is tied with the binding of NADH to enzymes. In particular, lower NAD(H) production is associated with a lower fraction of enzyme-bound NADH (higher *g* values), whereas a shift toward higher NAD(H) production is associated with a higher fraction of enzyme-bound NADH (lower *g* values). While a strong correspondence between fluorescence lifetimes and NADH activity is observed here, we note that it is possible that other autofluorescent molecules produced by P. aeruginosa contribute to the fluorescence lifetime measurements. The results here suggest that NADH is the predominant determinant of fluorescence lifetime in our experiments.

10.1128/mBio.02730-18.2 FIG S2 Fluorescence lifetimes of pyocyanin and pyoverdine mutants, purified pyocyanin and pyoverdine molecules, and wild-type cells treated with antimycin A. (A and B) The fluorescence lifetime phasor plots (left) and corresponding *g* values (right) of planktonic pyocyanin (Δ*phz1/2*) mutants (A) or pyoverdine (*pvdA*) mutants (B) cultured using 0.2% citrate or glucose as the sole carbon source. (C) Phasor plot indicating the fluorescence lifetimes of purified pyoverdine and pyocyanin. The dashed line indicates the metabolic trajectories of surface-attached (red) and planktonic (blue) P. aeruginosa observed in Fig. 2E and Fig. S1D, respectively. (D) Phasor plot (left) and corresponding *g* values (right) of planktonic wild-type P. aeruginosa cultured in 0.2% citrate as the sole carbon source and treated with 0.1% ethanol (control) or 10 μM antimycin A dissolved in 0.1% ethanol. Points on the phasor maps represent the average fluorescence lifetime of approximately five cells. The center and axis length of each ellipse corresponds to the mean and standard deviation, respectively, of all cells pooled from three independent experiments. The bars and error bars in the bar graph indicate the means and standard deviations of the means from three independent experiments, respectively. Planktonic P. aeruginosa cells were cultured in modified minimal medium and harvested at mid-exponential phase at an OD_600_ of 0.2. *, *P* < 0.05; ***, *P* < 0.001. Full statistical details are given in Table S1. Download FIG S2, PDF file, 0.1 MB. Copyright © 2020 Perinbam et al. 2020 Perinbam et al. This content is distributed under the terms of the Creative Commons Attribution 4.0 International license.

### A growth transition accompanies virulence induction.

**FIG 3:**
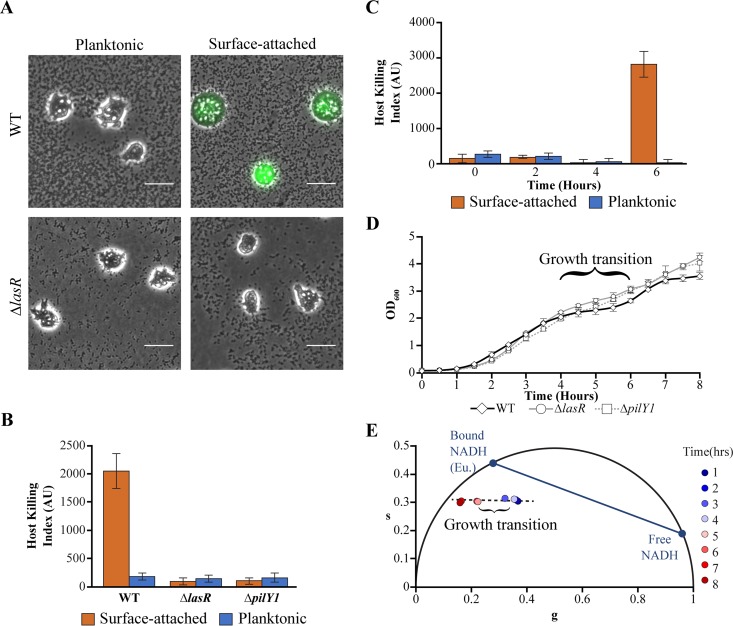
Virulence activation coincides with a growth transition. (A) An image-based assay using amoeba host cells and calcein-AM fluorescence (green) indicates the virulence of wild-type (WT) P. aeruginosa or P. aeruginosa Δ*lasR* and is used to compute host killing indexes. Bars, 25 μm. (B) Host killing indexes (in arbitrary units [AU]) for planktonic or surface-attached subpopulations of P. aeruginosa (wild-type, Δ*lasR*, and Δ*pilY1*) following 6 h of growth from dilution of a saturated culture. (C) The host killing indexes of planktonic or surface-attached wild-type P. aeruginosa following 0, 2, 4, and 6 h of growth. (D and E) Growth profiles measured using optical density (OD_600_) (D) and fluorescence lifetimes of planktonic P. aeruginosa (E). For panels B through D, bars and data points represent the average values for three independent experiments, and error bars indicate standard deviations. P. aeruginosa were cultured in rich (PS:DB) medium.

We characterized changes in fluorescence lifetime during the onset of virulence. Virulence was measured using an image-based assay using amoebae as host cells (12, 50). This assay is sensitive to virulence-activated and low-virulence phenotypes but does not capture intermediate virulence phenotypes (50). P. aeruginosa bacteria that are attached to a rigid substrate transition from a low-virulence state to a virulence-activated state during late exponential phase (12, 13). We confirmed that surface-attached P. aeruginosa cells activate virulence in our growth conditions in rich medium using amoebae as host cells and a calcein acetoxymethyl (calcein-AM) stain, which fluoresces when amoebae are stressed (12, 50) (Fig. 3A and B). We also confirmed that surface-activated virulence requires the master quorum-sensing regulator LasR and the surface sensing-associated protein PilY1 (12, 21) (Fig. 3A and B; see also Fig. S3A in the supplemental material). The mutations decrease the density of P. aeruginosa on the surface (Fig. S3B). However, we do not attribute the changes in virulence to the decrease in surface density, as the roles of LasR and PilY1 in regulating surface-activated virulence have been established previously (12).

**FIG 4:**
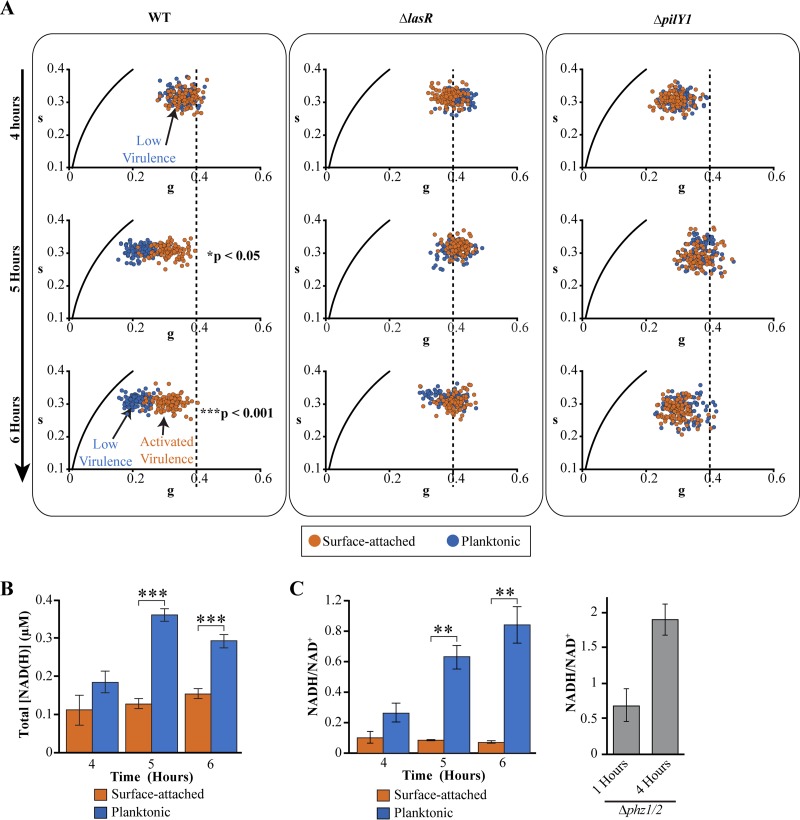
Virulence-activated and low-virulence populations have distinct fluorescence lifetimes and NAD(H) concentrations. (A) Fluorescence lifetime phasor maps of surface-attached (red) or planktonic (blue) populations of wild-type (left panel), Δ*lasR* (middle panel), or Δ*pilY1* (right panel) P. aeruginosa during the growth transition phase. Each data point represents the value for a single P. aeruginosa cell. Data are pooled from three independent experiments. A vertical dashed line at a *g* value of 0.4 is plotted for reference. (B and C) Total NADH and NAD^+^ concentrations (B) and the corresponding NADH/NAD^+^ ratios (C) for surface-attached and planktonic wild-type P. aeruginosa during the growth transition phase. Bars indicate the mean values from three independent experiments and error bars represent standard deviations. P. aeruginosa cells were cultured in rich (PS:DB) medium, with the exception of the Δ*phz1/2* strain, which was cultured in modified MOPS medium. Values that are significantly different are indicated by bars and asterisks as follows: **, *P* < 0.01; ***, *P* < 0.001. Full statistical details are given in Table S1 in the supplemental material.

10.1128/mBio.02730-18.3 FIG S3 Host killing indexes and surface densities of wild-type, quorum sensing-defective, and surface sensing-defective P. aeruginosa, and fluorescence lifetime *g* values and NADH and NAD^+^ concentrations of planktonic and surface-attached P. aeruginosa during the growth transition. (A) Host killing indexes of surface-attached wild-type, Δ*lasR*, or Δ*pilY1* P. aeruginosa that were assayed for virulence after 0 to 6 h of growth following dilution from a saturated culture. Host-killing indexes were determined using amoebae as host cells. Bars indicate the averages from three independent experiments, and error bars indicate standard deviations. (B) Corresponding surface densities of wild-type, Δ*lasR*, or Δ*pilY1* P. aeruginosa after 6 h of growth following dilution from a saturated culture. Bars indicate the averages of three surface density measurements, and error bars indicate standard deviations. (C) The fluorescence lifetime *g* values of surface-attached (red) or planktonic (blue) wild-type, Δ*lasR*, or Δ*pilY1* P. aeruginosa at 4, 5, or 6 h following dilution from an overnight culture, using the same data set shown in Fig. 4A. Bars and error bars indicate the means and standard deviations, respectively, of the means from three independent experiments. (D and E) NADH and NAD^+^ concentrations of surface-attached (D) or planktonic (E) P. aeruginosa during the same period. Bars and error bars indicate the means and standard deviations from three independent experiments, respectively. P. aeruginosa were cultured in PS:DB medium. *, *P* < 0.05; ***, *P* < 0.001. Full statistical details are given in Table S1. Download FIG S3, PDF file, 0.1 MB. Copyright © 2020 Perinbam et al. 2020 Perinbam et al. This content is distributed under the terms of the Creative Commons Attribution 4.0 International license.

To identify the growth period during which virulence is induced, we measured virulence at 2-h intervals following dilution from an overnight saturated culture. Virulence was induced in surface-attached cells during a critical period between 4 to 6 h of growth (Fig. 3C). Interestingly, this critical period was marked by a growth transition that punctuated two distinct periods of exponential growth, as measured by the optical density of planktonic cells from cultures in flasks (Fig. 3D; described in Text S1 in the supplemental material). The growth transition is consistent with a diauxic shift in which the cellular growth rate is temporarily reduced while the enzymes required for utilizing a different metabolic pathway are produced (51). Growth transitions were observed in LasR and PilY1 mutants, which have low virulence, although the transition effects were significantly diminished in these strains (Fig. 3D). To determine whether central metabolic activity is altered during the growth transition, we measured the fluorescence lifetimes of planktonic cells from cultures in flasks. We observed a significant shift in fluorescence lifetimes between 4 and 5 h, coinciding with the growth transition, toward smaller *g* values (Fig. 3E). These results suggest that P. aeruginosa undergoes a significant metabolic rearrangement during the virulence induction period.

10.1128/mBio.02730-18.8 TEXT S1 Supplemental methods. Additional details on growth conditions, measurements of growth profiles, fluorescence lifetime imaging microscopy conditions, measurement of growth, supplementation with carbon sources or antimycin A to surface-attached cells, surface cell density measurements, and the classifier model. Download Text S1, PDF file, 0.09 MB. Copyright © 2020 Perinbam et al. 2020 Perinbam et al. This content is distributed under the terms of the Creative Commons Attribution 4.0 International license.

### Virulence-activated and low-virulence populations are metabolically distinct.

Planktonic and surface-attached populations develop distinct virulence phenotypes during the growth transition period. At the start of the transition period at 4 h, both populations are in a low-virulence state (Fig. 3C and D). By the end of the transition period, surface-attached cells are induced for virulence, whereas planktonic cells remain in the low-virulence state (Fig. 3C and D). We hypothesized that planktonic and surface-attached cells undergo distinct metabolic changes during the growth transition period.

We monitored the fluorescence lifetimes and NAD(H) production in both populations from the same culture during the growth transition between 4 and 6 h. At the start of the growth transition (4 h following inoculation), the fluorescence lifetime profiles of the two populations were indistinguishable and had comparable *g* values (Fig. 4A, top left; see also Fig. S3C in the supplemental material). Two hours following entry into the growth transition (6 h following inoculation), in which P. aeruginosa enters a new period of exponential growth (Fig. 3D), the two populations exhibited distinct metabolic profiles (Fig. 4A, bottom left, and Fig. S3C). Surface-attached cells shifted to a smaller *g* value of 0.3, whereas planktonic cells shifted toward a significantly smaller *g* value of 0.2 (Fig. 4A, bottom left, and Fig. S3C). Measurements using the enzyme-cycling assay indicated that the subpopulations produced distinct concentrations of NAD(H) after 2 h (Fig. 4B), with the surface-attached population producing significantly lower levels of total NAD(H). This effect was due primarily to lower levels of NADH in the surface-attached population (Fig. S3D and E). Consequently, the NADH/NAD^+^ ratios in surface-attached P. aeruginosa were significantly lower than those in the planktonic population (Fig. 4C).

These results indicate that surface-attached and planktonic populations have distinct *g* values, total NAD(H) concentrations, and NADH/NAD^+^ ratios toward the end of the growth transition. In surface-attached P. aeruginosa, the decreased NAD(H) production, lower NADH/NAD^+^ ratios, and greater abundance of free NADH, as indicated by the FLIM measurements, are consistent with a less active metabolic state compared to the planktonic population. These results suggest that planktonic and surface-attached P. aeruginosa enter the growth transition period with identical metabolic states but exit the period with distinct metabolic activities, with the planktonic population having greater NADH-associated metabolic activity than the surface-attached population.

Surface-attached and planktonic cells have distinct host-killing activities at the end of the growth transition, with surface-attached and planktonic cells exhibiting virulence-activated or low-virulence states, respectively (Fig. 3C). Coupled with the observation that surface-attached and planktonic populations have distinct metabolic states, these observations suggest a link between metabolic states and virulence (Fig. 4A). We investigated whether metabolic activity is altered in LasR and PilY1 mutants, which have significantly reduced host-killing activity (12, 15, 16, 19–21). Surface-attached or planktonic populations of these mutants do not kill amoebae (Fig. 3A and B; see also Fig. S3A in the supplemental material). We found that the FLIM *g* values of both surface-attached and planktonic populations of these mutants were indistinguishable during the growth transition (Fig. 4A). In particular, we note the higher baseline *g* value of 0.4 in the LasR mutant, which suggests a higher abundance of free NADH in this strain. The LasR mutant is impaired in the production of the pyocyanin (52), which functions as a major electron acceptor and reacts with NADH (46). The shift to a higher *g* value in the LasR mutant may reflect a lack of interaction between NADH and pyocyanin.

### Perturbation of central metabolism alters virulence activation.

**FIG 5:**
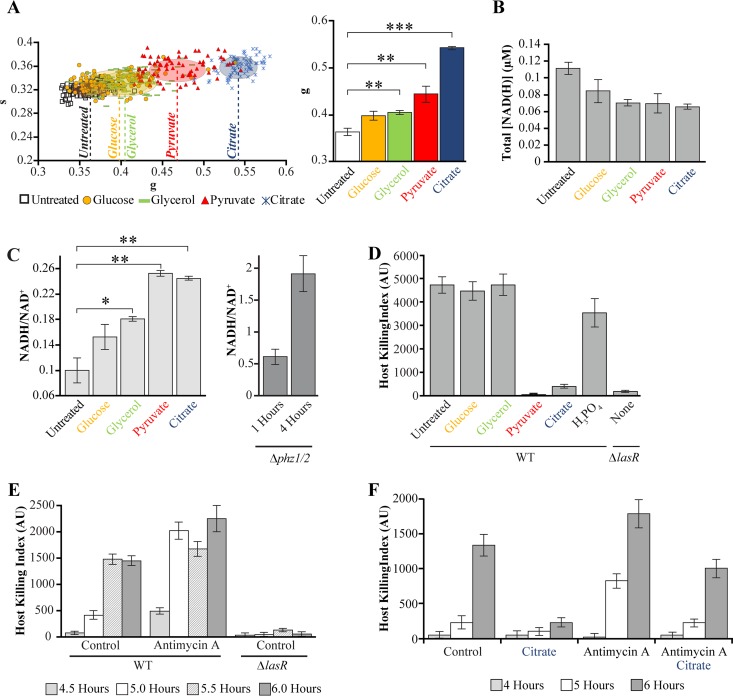
Perturbing central metabolism inhibits or induces earlier activation of virulence. (A) Fluorescence lifetime phasor plot (left) and *g* values (right) of surface-attached P. aeruginosa cultured in rich (PS:DB) medium, supplemented with carbon sources at 0.2% concentration at 3 h following dilution, and harvested after an additional hour of growth. Each data point represents the value for a single P. aeruginosa cell. The center and axis length of each ellipse corresponds to the mean and standard deviation, respectively, of all cells pooled from three independent experiments. The bars and error bars in the bar graph indicate the means and standard errors of the means of three independent experiments, respectively. (B and C) Total NADH and NAD^+^ concentrations (B) and corresponding NADH/NAD^+^ ratios (C) for surface-attached P. aeruginosa cultured under conditions identical to those in panel A. (D) Host killing indexes for surface-attached wild-type or Δ*lasR* P. aeruginosa assessed after 6 h of growth. Wild-type cultures were supplemented after 3 h of growth with individual carbon sources or phosphoric acid, at 0.2% concentration. Bars are the averages for three independent experiments, and error bars represent standard deviations. (E) Host killing indexes of wild-type or Δ*lasR* surface-attached P. aeruginosa at 4.5 to 6 h of growth following treatment at 3 h with 0.1% ethanol (control) or 10 μM antimycin A dissolved in 0.1% ethanol. (F) Host killing indexes of wild-type surface-attached P. aeruginosa following treatment at 3 h with 0.1% ethanol (control), 0.2% citrate, 10 μM antimycin A dissolved in 0.1% ethanol, or with 0.2% citrate and 10 μM antimycin A dissolved in 0.1% ethanol. All P. aeruginosa cells were cultured in rich (PS:DB) medium. Values that are significantly different are indicated by bars and asterisks as follows: *, *P* < 0.05; **, *P* < 0.01; ***, *P* < 0.001. Full statistical details are given in Table S1 in the supplemental material.

We investigated the extent that altered central metabolic activity affects host-killing activity in surface-attached cells. We cultured P. aeruginosa in rich medium and supplemented the cultures at the beginning of growth transition with carbon sources that mapped to distinct positions along the fluorescence lifetime trajectory (Fig. 2E) and that produced distinct changes in NAD(H) production (Fig. 2C). We observed that all carbon sources increased the FLIM *g* values, decreased NAD(H) production, and increased NADH/NAD^+^ ratios (Fig. 5A to C). The changes in NAD(H) production and NADH/NAD^+^ ratios were due primarily to decreases in NAD^+^ production (see Fig. S4A in the supplemental material). No change in NADH production was detected. Citrate, and pyruvate produced the greatest increases in *g* values, consistent with significant decreases in the relative abundance of enzyme-bound NADH, caused the greatest decreases in NAD(H) production, and caused the greatest increases in NADH/NAD^+^ ratio (Fig. 5A to C). In contrast, supplementation with glucose induced relatively small changes in FLIM *g* values, NAD(H) production, and NADH/NAD^+^ ratios (Fig. 5A to C). These results suggest that the treatment of surface-attached P. aeruginosa with carbon sources at the beginning of the growth transition induces the populations into metabolic states that have different levels of NADH activity.

10.1128/mBio.02730-18.4 FIG S4 Impact of supplementing carbon sources on NAD(H) concentrations and surface density in surface-attached P. aeruginosa. (A) NADH and NAD^+^ concentrations in surface-attached wild-type P. aeruginosa that have been supplemented with the indicated carbon sources at 0.2% concentration at 3 h following dilution from an overnight culture and cultured for an additional hour. P. aeruginosa cells were cultured in PS:DB. Bars indicate the averages from three experiments, and error bars indicate standard deviations. (B) Surface densities of P. aeruginosa from virulence assays after 6 h of growth. Bars indicate the averages for three surface density measurements, and error bars indicate standard deviations. Download FIG S4, PDF file, 0.07 MB. Copyright © 2020 Perinbam et al. 2020 Perinbam et al. This content is distributed under the terms of the Creative Commons Attribution 4.0 International license.

Analysis of host-killing activity revealed that citrate inhibited virulence in surface-attached P. aeruginosa (Fig. 5D). Similar results were observed using pyruvate, which had similar impacts on FLIM *g* values, NAD(H) production, and NADH/NAD^+^ ratios as citrate (Fig. 5A to C). In contrast, treatment with glucose or glycerol, which produced the smallest changes in NADH/NAD^+^ ratios and FLIM *g* values, had no effect on host-killing activity. The reduction in host-killing activity by citrate or pyruvate was not due to changes in the density of P. aeruginosa on surfaces, as the treatments did not decrease the surface density (see Fig. S4B in the supplemental material). The reduction in host-killing activity was also not due to changes in pH, as treatment with phosphoric acid at the same concentration had little impact on host-killing activity (Fig. 5D). These results suggest that a change in NAD(H) activity and enzyme-bound NADH fraction inhibits the activation of host-killing factors.

In separate experiments, we supplemented cultures at the beginning of the growth transition with antimycin A, which inhibits NADH oxidation. Treatment of surface-attached P. aeruginosa with antimycin A induced virulence 30 min earlier than untreated cells (Fig. 5E). This effect was inhibited by the cotreatment of antimycin A with citrate (Fig. 5F).

## DISCUSSION

Bacterial virulence is regulated by a number of factors that ensure successful infection. How the metabolic state of the cell changes during virulence induction has been unknown. Our results indicate that a shift in central metabolism, in the form of changes in NADH and NAD^+^ abundances and NADH binding to enzymes, accompanies the induction of virulence in P. aeruginosa. Using this finding, we perturb central metabolism to inhibit virulence or to induce virulence at an earlier time. As NADH is utilized as a central metabolic currency broadly across bacterial species, our results suggesting a role for NADH abundance in the regulation of virulence could have far-reaching significance.

We have established a metabolic trajectory in P. aeruginosa using the phasor approach to fluorescence lifetime imaging microscopy. We observed that positions along the *g* axis of the fluorescence lifetime trajectory negatively correlated with total NAD(H) production. Greater FLIM *g* values, indicating decreased enzyme-bound NADH within the cell, correlated with decreases in NAD(H) production. In addition, analysis of the cumulative fluorescence lifetime data using a K-means entropy clustering algorithm identified five distinct metabolic states into which P. aeruginosa cells can be clustered (see Fig. S5 in the supplemental material).

10.1128/mBio.02730-18.5 FIG S5 Clustering analysis of P. aeruginosa fluorescence lifetimes identifies distinct metabolic states. (A) Five distinct metabolic clusters (cluster 1 [C1] to cluster 5 [C5]) were identified using a K-means clustering algorithm using composite fluorescence lifetime data of P. aeruginosa cells from the current study and from a previous study (37). (B) Silhouette analysis on K-means clustering was used to identify clusters. (C) The cluster score was highest for five clusters. Download FIG S5, PDF file, 1.8 MB. Copyright © 2020 Perinbam et al. 2020 Perinbam et al. This content is distributed under the terms of the Creative Commons Attribution 4.0 International license.

By establishing fluorescence lifetime maps and performing NAD(H) concentration measurements and host-killing assays, we have measured the dynamics of central metabolism in P. aeruginosa during the activation of virulence. Our analysis revealed that P. aeruginosa undergoes a rapid and distinct metabolic rearrangement during the growth transition that differentiates cells into low-virulence or virulence-activated populations. At the beginning of the growth transition when P. aeruginosa entered a period of reduced growth rate, both planktonic and surface-attached populations were metabolically indistinguishable by FLIM and NAD(H) measurements. At the end of the transition, planktonic populations had an increased proportion of enzyme-bound NADH and increased the production of NAD(H) but did not activate host-killing factors. In contrast, surface-attached populations had comparatively less enzyme-bound NADH and decreased NAD(H) production, which resembled a state of metabolic dormancy, and transitioned to an activated virulence state.

The observation that virulent (surface-attached) populations are metabolically distinct from low-virulence (planktonic) populations raises the possibility that altering central metabolism activity could affect virulence activation. Treatment of surface-attached P. aeruginosa with citrate and pyruvate decreased the enzyme-bound NADH pool, decreased the total NAD(H) production, and abolished host-killing activity. In contrast, glucose and glycerol had relatively small impacts on the level of enzyme-bound NADH and NAD(H) production and had no effect on host-killing activity.

The impacts of individual carbon sources on host-killing activity may be interpreted in the context of the glyoxylate pathway, which bypasses the TCA cycle in favor of carbon preservation for gluconeogenesis and biomass production. The glyoxylate pathway activates the expression of type III secretion system and is important for lung infection models (53, 54). Growth in citrate and pyruvate in P. aeruginosa biases metabolic activity in favor of the TCA pathway and away from the glyoxylate pathway (55). Thus, the inhibition of virulence observed here could be explained by the inhibition of the glyoxylate pathway by citrate or pyruvate. Treatment of surface-attached P. aeruginosa using an oxidase inhibitor induced virulence at an earlier time, which was also inhibited by treatment of citrate. Glucose does not appear to inhibit the glyoxylate pathway, and glycerol is not expected to inhibit the pathway (55). Consistent with our interpretation, supplementation with glucose or glycerol had no impact on host-killing activity. Together, our results suggest a model in which the glyoxylate pathway is activated in surface-attached populations, which results in the expression of host-killing factors. In this model, the activation of the pathway can be inhibited by citrate or pyruvate, but not by glucose or glycerol. The observed changes in NAD(H) production and the fraction of enzyme-bound NADH may be indicative of changes in TCA and glyoxylate pathway utilization. The decreased production of NAD(H) in surface-attached populations is consistent with inactivation of the TCA cycle in favor of the glyoxylate pathway.

A recent preprint indicates that alkyl quinolines are responsible for cytotoxicity in surface-associated populations (16). Anthranilate is a metabolic precursor for quinolones (56), and its availability may have a significant impact on the production of these cytotoxic factors in surface-associated populations. The alteration of central metabolites could thus function as a regulator to rapidly coordinate virulence during the critical growth transition period.

Virulence is observed in our experiments only in surface-attached cells. Low-virulence planktonic cells produce greater levels of NAD(H) and have greater enzyme-bound NADH. The mechanisms that give rise to the distinct metabolic states are unclear. The availability of electron acceptors and surface sensing in P. aeruginosa could have an impact on metabolism. These results thus suggest an important role for electron transfer activity in the activation of virulence mechanisms. Future experiments will need to address the extent to which NAD(H) production and free NADH in planktonic cells impact the production of host-killing factors.

Fluorescence lifetime imaging microscopy provides spatial measurements of metabolism and may be a useful tool for measuring metabolic activity across multiple length scales from single cells to mature biofilms. We observed that fluorescence lifetimes were spatially heterogeneous in the cytoplasm of P. aeruginosa, which is consistent with the subcellular localization of metabolic activity (57). Future experiments will need to address the impact of changes in central metabolism on the spatial organization of NADH activity. In addition, metabolic dormancy in biofilms is associated with antibiotic resistance (58). The use of FLIM to map spatial changes in metabolism in biofilms may thus open new avenues for the investigation of antibiotic resistance in biofilms.

Antivirulence therapy is a proposed strategy for combating pathogenesis as an alternative to conventional antibiotics, which typically target bacterial growth (59). The identification that NADH levels affect virulence induction highlights a potential target for virulence inhibition. Our results suggest metabolic manipulation as a strategy to inhibit virulence. Strategies such as targeting metabolic pathways involved in NAD(H) production or growth in the presence of bacteria that secrete metabolites that affect NAD(H) production could be effective at inhibiting virulence. Within microbiomes, complex microbial communities, and host environments, metabolite cross-feeding could have a significant impact on virulence activation in pathogens.

## MATERIALS AND METHODS

### Growth conditions and strains.

Pseudomonas aeruginosa strains were streaked onto LB-Miller (BD Biosciences, Franklin Lakes, NJ) petri dishes containing 2% Bacto agar (BD Biosciences) and incubated at 37°C to obtain single colonies. Individual colonies were inoculated into modified PS:DB, which is a rich medium that supports coculturing of P. aeruginosa cells with Dictyostelium discoideum (amoeba) (12). The modified PS:DB medium, hereafter referred to as PS:DB medium in this work, is the same formulation of PS:DB medium as described previously (12) except that PS medium was used at a concentration of 90% (vol/vol) instead of 10% (vol/vol). P. aeruginosa strains were cultured overnight in PS:DB medium in a rotary drum rotating at 24 rpm or orbital shaker rotating at 200 rpm and 37°C, diluted 1:100 into a plastic or glass dish containing the same medium, and cultured between 4 to 6 h. Alternatively, strains were cultured in minimal medium A (60) containing 0.2% glucose, diluted 1:100 into minimal medium A that was modified to exclude citrate (hereafter referred to as modified minimal medium A) and containing one of the following carbon sources at a concentration of 0.2%: glucose, glycerol, citrate, or pyruvate (Sigma, St. Louis, MO), and cultured to an optical density at 600 nm (OD_600_) of 0.2 at 37°C.

D. discoideum was grown axenically in PS medium at 22°C as described previously (12) and harvested for virulence assays when cultures reached an OD_600_ between 0.2 to 0.5.

Wild-type P. aeruginosa strain PA14 (61), PA14 strains containing a Δ*lasR* (AFS20.1) (62) or Δ*pilY1* (19) deletion, a *pvdA*::Mar2xT7 mutation (63), or the Δ*phzA1-G1* Δ*phzA2-G2* mutations (64, 65) (original strain name DKN330, referred to here as Δ*phz1/2*), and the D. discoideum strain AX3 (66) were used for these experiments.

### Fluorescence lifetime imaging microscopy (FLIM).

Fluorescence lifetime measurements were performed using a custom-built multiphoton microscope setup based on an Olympus FV1000 system and an Olympus IX81 microscope (Olympus, Waltham, Massachusetts) as described previously (67). The FLIM microscope uses an 80 MHz ultrafast Ti:Sapphire Mai Tai laser (Spectra-Physics, Santa Clara, CA) set at 740 nm for multiphoton excitation. The setup used a 690-nm SP dichroic 460/80-nm filter pair for separating emission and a PlanApo N Olympus oil immersion 60× (1.42-numerical-aperture [NA]) objective (Olympus, Waltham, MA), which is capable of bacterial single-cell resolution imaging. An H7422P-40 photomultiplier tube module (Hamamatsu, Bridgewater, NJ) and A320 FastFLIM Box (ISS, Champaign, IL) were used to measure fluorescence lifetime. Image acquisition was controlled by SimFCS software version 4 (64-bit) (Laboratory for Fluorescence Dynamics, Irvine, CA). Planktonic and surface-attached cells were isolated by modifying a protocol described previously (12, 50) (see Fig. S6A in the supplemental material).

10.1128/mBio.02730-18.6 FIG S6 Schematic summarizing fluorescence lifetime analysis and the host-killing assay. (A) Planktonic or surface-attached P. aeruginosa cells are isolated from the same culture grown in a petri dish, immobilized using an agar pad, and imaged using a fluorescence lifetime microscope. (B) Individual cells or cell clusters are segmented using fluorescence intensity images and an intensity threshold. Fluorescence lifetimes in individual cells or cell clusters are transformed to a two-dimensional phasor plot containing *s* and *g* axes. (C) Planktonic or surface-attached P. aeruginosa cells are isolated from the same culture grown in a petri dish, mixed with axenically grown amoeba host cells, immobilized with an agar pad containing calcein-AM, and imaged using a fluorescence microscope. Download FIG S6, PDF file, 0.2 MB. Copyright © 2020 Perinbam et al. 2020 Perinbam et al. This content is distributed under the terms of the Creative Commons Attribution 4.0 International license.

Masks for bacterial cells were created through SimFCS using fluorescence intensity images and image intensity thresholding (see Fig. S6B in the supplemental material). Fluorescence lifetimes within the masked areas were transformed using $g_{i,j}(\text{ω})=\int_{0}^{\infty }I_{i,j}(t)\cos(\text{ω}t)\mathrm{dt}/\int_{0}^{\infty }I_{i,j}(t)\mathrm{dt}$ and $s_{i,j}(\text{ω})=\int_{0}^{\infty }I_{i,j}(t)\sin(\text{ω}t)\mathrm{dt}/\int_{0}^{\infty }I_{i,j}(t)\mathrm{dt}$, where *I*(*t*) is the fluorescence intensity decay, ω is the laser repetition angular frequency, and the indexes *i* and *j* identify a pixel of the image.

The microscope was calibrated before each session by setting the fluorescence lifetime obtained for 10 μM rhodamine 110 (Sigma, St. Louis, MO) to 4.0 ns. The laser power was set at 20% (<3 mW at the back aperture of the microscope) using an acousto-optic modulator (AA Opto Electronic, Orsay, France). Subcellular fluorescence lifetimes (Fig. 1A) were collected using the 20× digital zoom mode at a rate of 1.7 s/frame. All other P. aeruginosa measurements were performed using the 6× digital zoom mode using the same frame rate. For all measurements, 40 sequential frames were acquired to generate a single FLIM image.

### Virulence assay.

P. aeruginosa cells were assayed using an image-based virulence assay as described previously (12, 50). P. aeruginosa strains were cultured in PS:DB medium, diluted 1:100 into plastic petri dishes (60 by 15 mm) (Corning, Corning, NY), cultured for 4 to 6 h by shaking at 100 rpm, and harvested. Planktonic cells were assayed by transferring 10-μl volume of culture from the petri dish to a new petri dish, mixing with an equal volume of D. discoideum that had been grown to an OD_600_ of 0.2 to 0.5, and immobilizing by placing an agar pad on top of the mixture (see Fig. S6C in the supplemental material). Agar pads were made using Bacto agar at a concentration of 1%, DB buffer, and 1 μM calcein acetoxymethyl (calcein-AM) ester (Molecular Probes, Eugene, OR) and were cut into 1.5- by 1.5-cm squares (12, 50). Surface-attached cells were assayed by aspirating planktonic cells from the culture, washing with DB buffer to remove planktonic cells, mixing with an equal volume of D. discoideum, and immobilizing by placing an agar pad on top of the mixture (Fig. S6C) (50). The immobilized planktonic or surface-attached P. aeruginosa and D. discoideum were incubated at room temperature for 1 h and imaged using fluorescence microscopy. The host killing index (12, 50) was computed as the average ratio of calcein-AM fluorescence to cell area in individual amoeba cells for 30 to 200 cells in each experiment.

Fluorescence microscopy to assess calcein-AM fluorescence was performed using a Nikon Eclipse Ti-E microscope (Nikon, Melville, NY) containing Nikon 10× Plan Fluor Ph1 (0.3-NA) and 20× S Plan Fluor Nikon (0.45-NA) objectives, a Sola light engine (Lumencor, Beaverton, OR), an LED-DA/FI/TX filter set (Semrock, Rochester, NY) containing a 409/493/596 dichroic and 474/27-nm and 525/45-nm filters for excitation and emission, respectively, and a Hamamatsu Orca Flash 4.0 V2 camera (Hamamatsu, Bridgewater, NJ). Images were acquired using Nikon NIS-Elements and analyzed using custom-built software written previously (12) in Matlab (Mathworks, Natick, MA).

### NAD (NADH, NAD^+^) concentrations.

The concentrations of reduced and oxidized NAD (NADH, NAD^+^) were measured using a colorimetric enzyme-cycling assay as described previously (45). P. aeruginosa strains were cultured overnight in PS:DB medium, diluted 1:100 into plastic petri dishes (60 by 15 mm) (Corning, Corning, NY) containing the same medium, and grown for 4 to 6 h. Alternatively, strains were cultured overnight in minimal medium, diluted 1:100 into plastic petri dishes containing modified minimal medium with single carbon sources, and grown to an OD_600_ of 0.2. Cultures were harvested in a glove box chamber (Bel-Art, Wayne, NJ) that had been vacuumed to remove air and flushed with nitrogen constantly throughout the experiment to prevent oxidation of NADH. Subsequent steps were performed, and solutions were prepared in the glove box unless otherwise indicated. Solutions containing 0.05 to 1 μM of NADH (Sigma) or NAD^+^ (Sigma) were included as calibration controls.

Planktonic cells were isolated from petri dishes and pelleted by centrifugation at 16,000 × *g* for 1 min, the residual supernatant was discarded, and the pellet was immediately resuspended in 0.2 M NaOH or HCl to extract NADH or NAD^+^, respectively, as described previously (45, 46). Surface-attached cells were harvested by aspirating planktonic cells from petri dishes, washing with DB buffer or modified minimal medium with no carbon source for strains cultured in PS:DB medium or modified minimal medium A containing single carbon sources, respectively, adding 0.2 M NaOH or HCl, and scraping surfaces with a cell scraper (Sarstedt, Nümbrecht, Germany). For a control, the Δ*phz1/2* strain was cultured overnight in modified morpholinepropanesulfonic acid (MOPS) synthetic medium (46), diluted 1:100 in the same medium in culture tubes for 1 to 4 h, pelleted by centrifugation, and resuspended in 0.2 M NaOH or HCl, as described previously (46). The resuspensions in NaOH or HCl were neutralized using equal volume of 0.1 M HCl or NaOH, respectively, and portions were assayed for protein content, NADH, and NAD^+^. Resuspensions were assayed for protein content using a BCA assay kit (ThermoFisher Scientific, Waltham, MA) using a 50:1 mixture of solutions A and B from the kit. Solutions were incubated at 37°C for 30 min and measured for absorbance at 562 nm. Resuspensions that were not used for the BCA assay were normalized by protein content by diluting in Millipore filtered water.

NADH and NAD^+^ concentrations were determined by following the protocol described previously (45). Briefly, normalized resuspensions were centrifuged at 16,000 × *g* for 10 min at 4°C to remove cell debris and mixed with a reaction mixture containing 40 mM EDTA, 1 M bicine (Sigma), 4.2 mM 3-(4,5-dimethylthiazol-2-yl)-2,5-diphenyltetrazolium bromide (Sigma), 16.6 mM phenazine ethosulfate (Sigma), 1 mg/ml alcohol dehydrogenase (Sigma), and ethanol, and the absorbance at 570 nm of the solution was measured in a 96-well plate (Corning, Corning, NY) at 30°C every 30 s for 30 min using a BioTek Synergy HTX reader (BioTek, Winooski, VT). The reaction mixture and the normalized resuspensions were aliquoted and mixed inside the glove box. The centrifugation and absorbance measurements were performed outside the glove box. The absorbance per unit time was determined by fitting data on the initial velocity of enzyme activity, acquired between 2 and 10 min of the absorbance measurements. The absorbances per unit time for the NADH and NAD^+^ calibration controls were linearly fit for the full range of calibration concentrations to establish a linear equation that relates the absorbance per unit time and NAD(H) concentration. The NAD(H) concentrations of the planktonic and surface-attached cell resuspensions were determined by inputing the respective absorbance per unit time fits of the samples into these equations.

### Statistical analysis.

The significance of changes between experimental conditions was determined by using unpaired two-tailed heteroscedastic *t* tests. The *t* values, degrees of freedom, and *P* values are given in Table S1 in the supplemental material. For fluorescence lifetime phasor plots, the center of ellipses indicates the mean of all cells pooled from multiple experiments. The axis lengths of the ellipses indicate the standard deviation of the pooled data set. Unless otherwise indicated, bars and error bars in bar graphs indicate the mean and standard deviation of the means from multiple independent experiments, respectively.

Further details on growth conditions, measurements of growth profiles, FLIM measurements, surface density measurements, and the classifier model are described in Text S1 in the supplemental material.
