# Supplementary material for: A Shift in Central Metabolism Accompanies Virulence Activation in Pseudomonas aeruginosa
Source: mBio. 2020 Mar 10;11(2):e02730-18. doi: 10.1128/mBio.02730-18 (PMC7064766; doi:10.1128/mBio.02730-18)
Supplement: TABLE S1 [file mBio.02730-18-st001.pdf]

Table S1

Figure	Comparison	t value	Degrees of freedom	p value
2B	Citrate, Glycerol	2.56	2	1.25E-01
4A	Surface-attached, planktonic (WT, 5 hrs)	8.17	2	1.47E-02
4A	Surface-attached, planktonic (WT, 6 hrs)	25.70	3	1.29E-04
4B	Surface-attached, planktonic (WT, 5 hrs)	23.93	4	1.81E-05
4B	Surface-attached, planktonic (WT, 6 hrs)	12.06	4	2.71E-04
4C	Surface-attached, planktonic (WT, 5 hrs)	12.62	2	6.22E-03
4C	Surface-attached, planktonic (WT, 6 hrs)	11.13	2	7.98E-03
5A	Untreated, glycerol	8.08	3	3.96E-03
5A	Untreated, pyruvate	6.29	3	8.12E-03
5A	Untreated, citrate	36.86	3	4.39E-05
5C	Untreated, glycerol	7.29	2	1.83E-02
5C	Untreated, pyruvate	13.51	2	5.43E-03
5C	Untreated, citrate	13.01	2	5.86E-03
S1A	Glycerol, citrate	4.97	3	1.56E-02
S1B	Glycerol, citrate	3.08	4	3.69E-02
S2A	Citrate, glucose	3.38	3	4.30E-02
S2B	Citrate, glucose	14.92	4	1.18E-04
S2D	Untreated, antimycin A	14.11	3	7.71E-04
S3C	Surface-attached, planktonic (WT, 5 hrs) (same as Fig. 4A)	8.17	2	1.47E-02
S3C	Surface-attached, planktonic (WT, 6 hrs) (same as Fig. 4A)	25.70	3	1.29E-04
