# Supplementary figures and images for: A Shift in Central Metabolism Accompanies Virulence Activation in Pseudomonas aeruginosa
Source: mBio. 2020 Mar 10;11(2):e02730-18. doi: 10.1128/mBio.02730-18 (PMC7064766; doi:10.1128/mBio.02730-18)

Figure S1

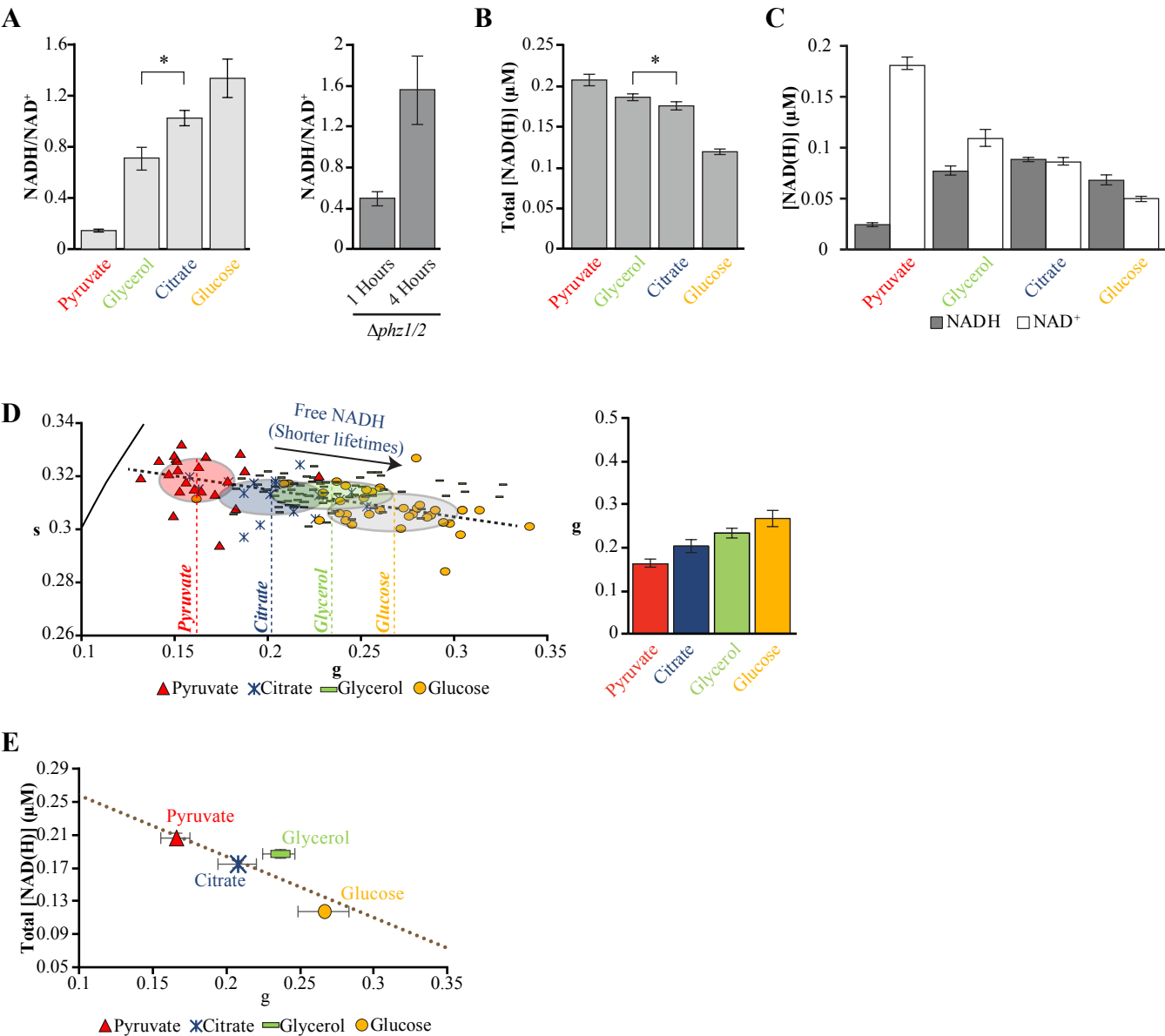

Supplement: FIG S1 [file mBio.02730-18-sf001.pdf]

**Figure S2**

**A**

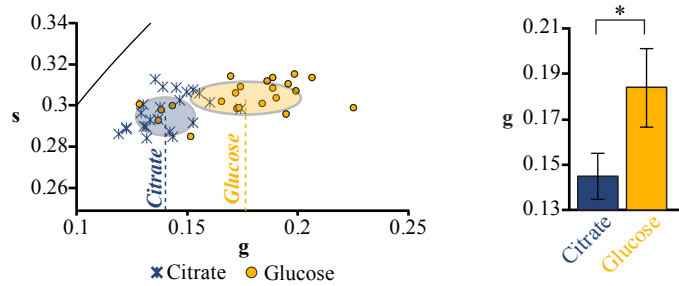

**B**

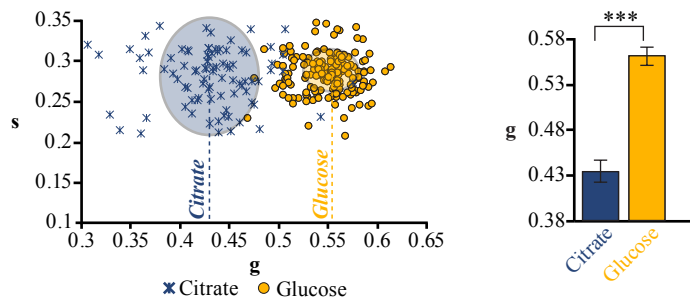

**C**

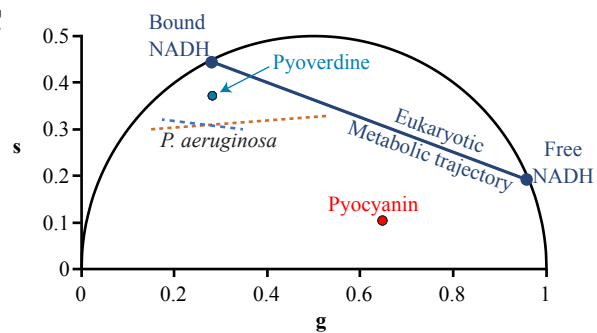

**D**

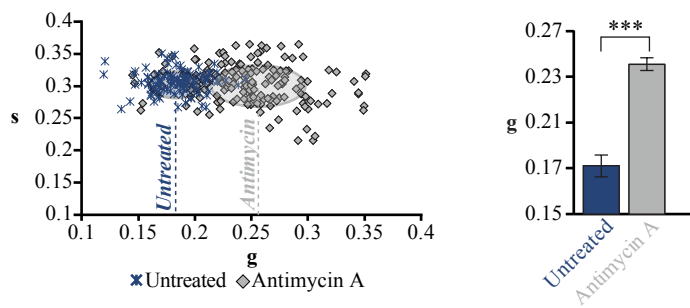

Supplement: FIG S2 [file mBio.02730-18-sf002.pdf]

**Figure S3**

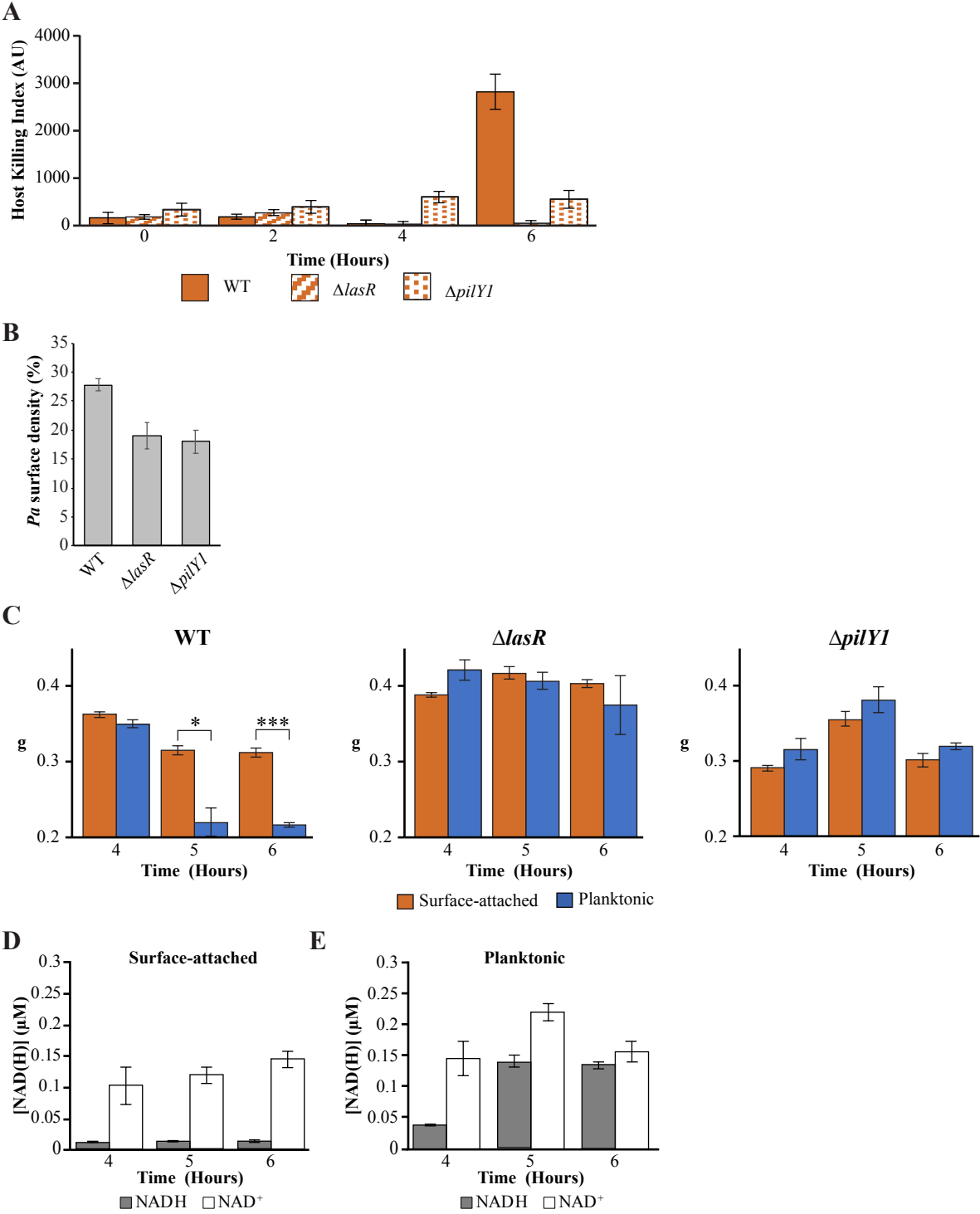

Supplement: FIG S3 [file mBio.02730-18-sf003.pdf]

**Figure S4**

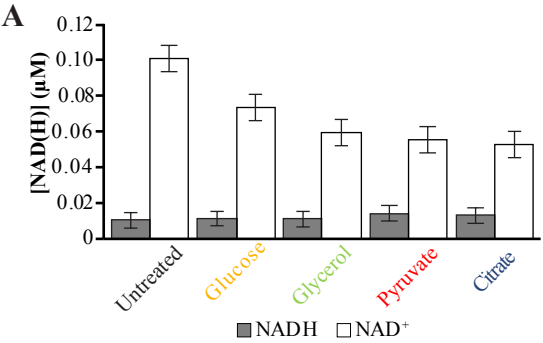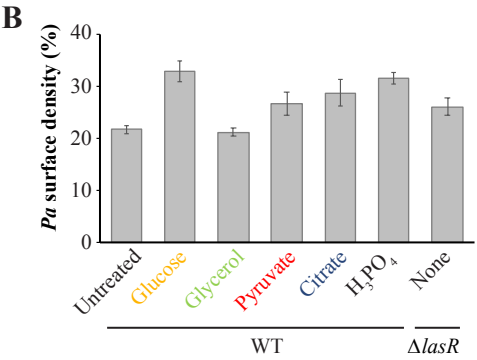

Supplement: FIG S4 [file mBio.02730-18-sf004.pdf]

**Figure S5**

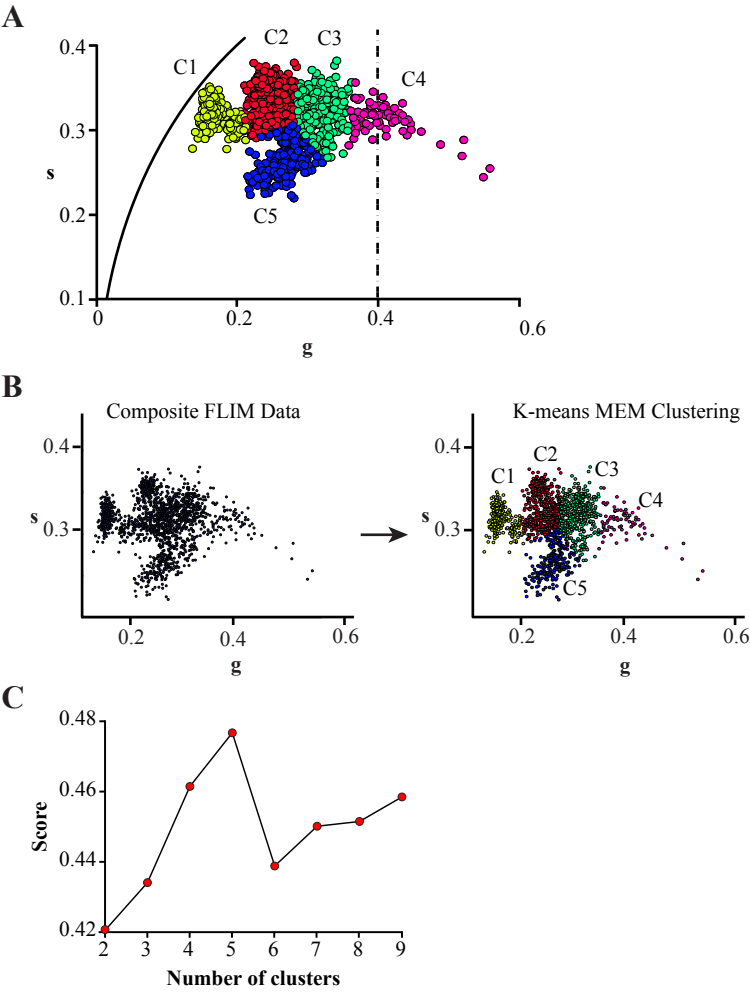

Supplement: FIG S5 [file mBio.02730-18-sf005.pdf]

**Figure S6**

**A**

**Fluorescence lifetime assay**

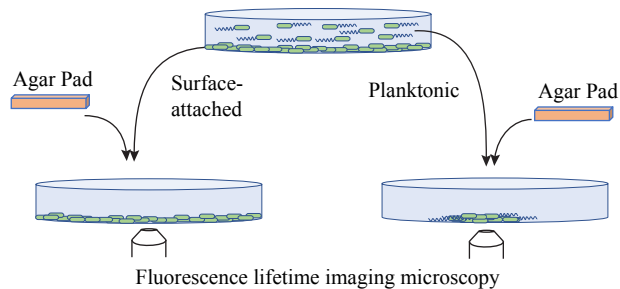

**B**

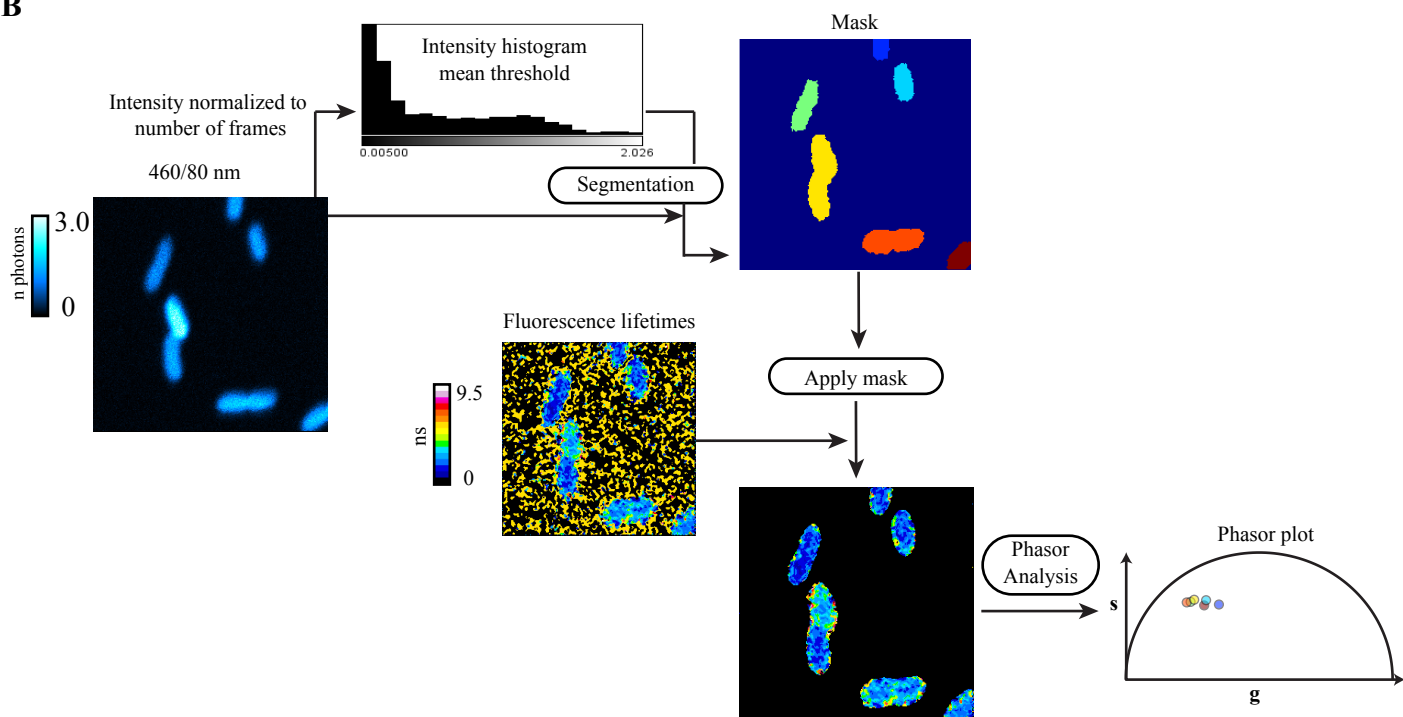

**C**

**Virulence assay**

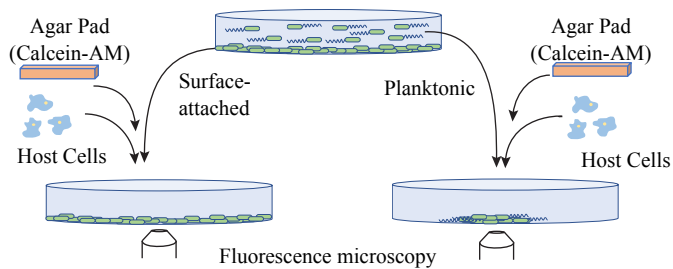

Supplement: FIG S6 [file mBio.02730-18-sf006.pdf]
