# Supplementary material for: A Shift in Central Metabolism Accompanies Virulence Activation in Pseudomonas aeruginosa
Source: mBio. 2020 Mar 10;11(2):e02730-18. doi: 10.1128/mBio.02730-18 (PMC7064766; doi:10.1128/mBio.02730-18)
Supplement: TEXT S1 [file mBio.02730-18-s0001.pdf]

SUPPLEMENTAL METHODS

Growth conditions

Citric acid or pyruvic acid (Sigma, St. Louis, MO) were used as carbon sources in experiments involving measurements of NAD(H) concentrations. Sodium citrate or sodium pyruvate were used in experiments involving FLIM measurements of planktonic cells, except for Fig. S2D, for in which citric acid was used. Planktonic and surface-attached cells were isolated by modifying a protocol described previously (1, 2).

Fluorescence lifetime imaging microscopy conditions

P. aeruginosa strains were cultured overnight in PS:DB, diluted 1:100 into 35 mm glass-bottom dishes (MatTek Corporation, Ashland, MA) containing the same medium, and cultured for 4 to 6 hours (Fig. 2E-F, 4A, 5A, and S3C). Alternatively, strains were cultured overnight in minimal medium A, diluted 1:100 into modified minimal media A with single carbon sources in culture tubes (Fig. S1D-E and S2A-B, D) or glass-bottom dishes and cultured to an OD_{600} of 0.2 (Fig. 2E-F).

Planktonic cells were transferred to a new glass-bottom dish, immobilized using an agar pad, and imaged immediately (Fig. S6A). Agar pads were made using 1% Bacto agar (BD, Franklin Lakes, NJ) and DB buffer (1, 2) or modified minimal medium A containing no carbon source for strains that were cultured in PS:DB or modified minimal medium, respectively, and were cut into 1.5 x 1.5 cm squares. Surface-attached cells were isolated by aspirating the supernatant from the dish, washing the dish to remove planktonic cells with DB buffer or modified minimal medium with no carbon source for strains cultured in PS:DB or modified minimal medium, respectively, and placing an agar pad on the dish surface, and were imaged immediately. Imaging was performed at room temperature.

For experiments involving FLIM measurements of the effects of antimycin A (Fig. S2D), *P. aeruginosa* were grown in culture tubes to saturation in modified minimal medium A containing 0.2 % citrate, diluted 1:100 into the same medium in culture tubes, supplemented with 10 μM antimycin A (Sigma, St. Louis, MO) with 0.1% ethanol or with 0.1% ethanol, cultured to an OD_{600} of 0.2, and immediately measured for fluorescence lifetime. Fluorescence lifetimes for pyocyanin and pyoverdine (Fig. S2C) were measured using solutions of 6.2 mM pyocyanin (P0046, Sigma, St. Louis, MO) in DMSO and 5 mg/mL pyoverdine (P8124, Sigma, St. Louis, MO) in deionized water, respectively. For Fig. S1D, cells with fluorescence intensities below a value of 1.0, as reported by the SimFCS software, were excluded from the analysis.

Measurement of growth profiles

The growth profiles of *P. aeruginosa* (Fig. 3D) were measured by diluting saturated overnight cultures to 1:100 in PS:DB into 50 mL of the same medium in a 250 mL Erlenmeyer flask, culturing at 37 ºC with shaking at 200 rpm, and measuring the OD_{600} of 1 mL of the culture every 30 mins using an Ultrospec10 spectrometer (Harvard Bioscience Inc., Holliston, MA). Cultures with OD_{600} measurements greater than 0.5 were diluted into fresh PS:DB and re-measured. The final OD_{600} was computed by normalizing the measurement by the dilution factor.

Supplementation with carbon sources or antimycin A to surface-attached cells

For experiments in which strains cultured in PS:DB were supplemented with individual carbon sources (Fig. 5A-D), pyruvic acid, citric acid, phosphoric acid, glycerol, or glucose was added to cultures at a final concentration of 0.2 % at 3 hours following a 1:100 dilution from an

overnight culture. Cultures were harvested after 1 additional hour of growth for measurements of fluorescence lifetime and NADH and NAD^+ concentrations. For experiments in which surface-attached strains that were cultured in PS:DB media were treated with antimycin A (Fig. 5E-F), 10 μM antimycin A dissolved in 0.1% ethanol, 0.1% ethanol, alone, or 10 μM antimycin A dissolved in 0.1% ethanol and 0.2 % citric acid were added after 3 hours of growth following a 1:100 dilution from an overnight culture. Host killing was measured after an additional 1 to 3 hours of growth (total of 4 to 6 hours of growth).

Surface density measurements

The density of *P. aeruginosa* cells on surface was measured as described previously (1) using amoebae cell viability phase contrast images that were acquired using a 10X or 20X objective. The IJ_Isodata algorithm (ImageJ 1.52q) was applied to phase contrast images to construct cell boundary masks. The cell density was computed by dividing the area covered by *P. aeruginosa* by the total area of the image.

Classifier model

K-means clustering was performed using the Scikit kmeans classifier (3) and minimum cluster entropy was estimated by maximizing the silhouette coefficient score (4) (Fig. S5).

SUPPLEMENTAL REFERENCES

1. Siryaporn A, Kuchma SL, O'Toole GA, Gitai Z. 2014. Surface attachment induces Pseudomonas aeruginosa virulence. Proc Natl Acad Sci 111:16860–16865.
2. Perinbam K, Siryaporn A. 2018. A Rapid Image-based Bacterial Virulence Assay Using Amoeba. J Vis Exp https://doi.org/10.3791/57844.
3. Pedregosa, F., Varoquaux, G, Gramfort, A, Michel, V, Thirion, B, Grisel, O, Blondel, M, Prettenhofer, P, Weiss, R, Dubourg, V, Vanderplas, J, Passos, A, Cournapeau, D, Brucher, M, Perrot, M, Duchesnay, E. 2011. Scikit-learn: Machine Learning in Python 12:2825–2830.
4. Rousseeuw PJ. 1987. Silhouettes: A graphical aid to the interpretation and validation of cluster analysis. J Comput Appl Math 20:53–65.
